# *Baccharis dracunculifolia* DC Hydroalcoholic Extract Improves Intestinal and Hippocampal Inflammation and Decreases Behavioral Changes of Colitis Mice

**DOI:** 10.1155/2022/5833840

**Published:** 2022-03-07

**Authors:** Tauani Caroline Santos França, Ana Julia Ribeiro, Luísa Natália Bolda Mariano, Ana Caroline dos Santos, Larissa Venzon, Lincon Bordignon Somensi, Ruan Kaio Silva Nunes, Camila André Cazarin, Karen Luz Okubo, Helenita Priscila Poerner, Jairo Kneupp Bastos, Márcia Maria de Souza, Luísa Mota da Silva

**Affiliations:** ^1^Postgraduate Program in Pharmaceutical Sciences, University of Vale Do Itajaí, Itajaí, Santa Catarina, Brazil; ^2^Postgraduate Program in Development and Society, Alto Vale Do Rio Do Peixe University, Caçador, Santa Catarina 89500-000, Brazil; ^3^School of Pharmaceutical Sciences of Ribeirão Preto, University of São Paulo, Ribeirão Preto, São Paulo, Brazil

## Abstract

The hydroalcoholic extract of B. dracunculifolia (HEBD) and its major compound p-coumaric acid were evaluated against the severity of intestinal inflammation and behavioral changes like depressive and anxious behavior in colitis mice. Colitis was induced in Swiss mice by oral dextran sulfate sodium (DSS) administration for five days. The mice received vehicle (10 ml/kg), HEBD (3, 30, or 300 mg/kg), or p-coumaric acid (15 mg/kg) orally, once a day for twelve days. Behavioral tests were performed on the 11^th^ and 12^th^ days after the beginning of the treatments. Moreover, the colon, cortex, and hippocampus were collected to analyze oxidative and inflammatory parameters. The treatment with HEBD (300 mg/Kg), but not p-coumaric acid, showed decreased disease activity index (DAI) values compared to the vehicle group and partially preserved the villi architecture and mucin levels. Furthermore, the HEBD increased the antioxidant defenses in the colon and hippocampus and reduced the myeloperoxidase activity and IL-6 levels in the colon from colitis mice. Colitis mice treated with HEBD did not show depressive-like behavior in the tail suspension test. HEBD reduced colon inflammation, while it maintains antioxidant defenses and mucin levels in this tissue. It may reduce neuropsychiatric comorbidities associated with colitis through its antioxidant effects.

## 1. Introduction

Ulcerative colitis (UC) is an idiopathic disease defined as a chronic colon and rectum inflammatory process. Despite the variety of existing drugs available for its treatment, it is estimated that 15–20% of the patients will require hospitalization [1]. The incidence and prevalence of UC differ by region, with an accelerated incidence rate in Western and newly industrialized countries [2]. The pathogenesis of this disease can be related to some environmental factors, oxidative stress, disruption of the epithelial barrier, dysregulation in the immune response, and impairment of the intestinal microflora [3].

The symptoms of UC may include weight loss, abdominal pain, diarrhea, rectal bleeding, and fatigue, and when the disease is limited to the rectum, the symptoms are more related to fecal incontinence and urgency of defecation. In severe cases, episodes of vomiting, fever, anorexia, and abdominal distension can occur [3, 4]. In addition to these symptoms, patients with UC are at higher risk of developing colorectal cancer [5] and are more likely to develop psychological conditions such as anxiety and depression and have impaired social interactions or careers [6].

Herbal medicine has become a frequent therapeutic resource in patients with UC [7]; therefore, the development of studies related to the pharmacological potential of medicinal plants becomes essential and a fertile field for increasing the therapeutic arsenal to the treatment of inflammatory bowel disease (IBD). In this context, Baccharis dracunculifolia DC (Asteraceae), known as “alecrim-do-campo,” a medicinal shrub native to Brazil, can be a natural resource in obtaining herbal medicines or phytopharmaceuticals for IBD treatment, especially UC.

Baccharis dracunculifolia has been described as the primary botanical source of Brazilian green propolis, responsible for many bioactive constituents of this propolis [8]. In addition, this Brazilian native plant has been widely used for medicinal purposes, including to treat gastrointestinal diseases, inflammation, and liver disorders [9, 10]. Regarding its anti-inflammatory potential, a few studies have described that B. dracunculifolia mitigates the inflammatory process in macrophages [11] and in rodents [12]. Furthermore, Cestari et al. [13] evaluated the intestinal anti-inflammatory of a B. dracunculifolia [14, 15] ethyl acetate extract in rats exposed to trinitrobenzene sulfonic acid (TNBS), a model of intestinal inflammation that triggers an immune response like Crohn disease in humans [16].

The pharmacological research about natural products could benefit current therapies for IBD or be integrated into conventional therapy, reducing the dosages of current therapies and their undesirable effects. Therefore, given the traditional use of B. dracunculifolia and the results from Cestari et al. [13], this study was performed to evaluate the efficacy of the hydroalcoholic extract of B. dracunculifolia (HEBD) against intestinal inflammation induced by oral intake of dextran sulfate sodium (DSS) in mice. It is noteworthy that the choice of this model reflects the fact that it triggers predominantly a TH2 immune response, which is prevalent in UC in humans [16–18]. In addition, the effect of p-coumaric acid, a major compound in HEBD, was evaluated on mice submitted to DSS-induced colitis, along with the effects of HEBD on behavioral changes in colitis mice. In this way, this study expands the knowledge about the pharmacological potential of HEBD in the treatment of UC in an adequate murine model.

## 2. Material and Methods

### 2.1. Plant Material and Extract Obtaining

Baccharis dracunculifolia aerial parts were collected in Ribeirão Preto at the University of São Paulo (USP) campus. The voucher specimen of B. dracunculifolia was identified by Milton Groppo Junior and deposited in the herbarium of the Department of Biology of Faculty of Philosophy, Science and Letters of Ribeirão Preto, University of São Paulo, Ribeirão Preto, SP, Brazil, under the number SPFR 06143. As previously described by Lemos et al. [9], the dried ground leaves (350 g) were extracted by maceration using (ethanol/water 7:3, v/v) exhaustively. The hydroalcoholic extract of B. dracunculifolia was concentrated under vacuum and lyophilized, furnishing 50.2 g (14.3%) of the crude hydroalcoholic extract (HEBD).

### 2.2. p-Coumaric Acid Isolation

The p-coumaric acid was isolated from HEBD with purity greater than 98%. For a detailed protocol of these procedures, see Lemos et al. [9] and Costa et al. [14]. In summary, the isolated compound was identified by spectroscopic analysis of ^1^H and 1^3^C NMR using deuterated solvents (chloroform CDCl_3_, dimethyl sulfoxide-DMSO-d6, and acetone-d6) in Bruker spectrometers (DRX-400 or DRX500), working at 400 or 500 MHz for ^1^H and at 100 or 125 MHz for 1^3^C. Tetramethylsilane was used as an internal reference. High-resolution mass spectral (MS) data were acquired using Thermo Scientific Exactive PlusTM H-ESI II Instrument with OrbitrapTM System at spray voltage 3.6 kV in negative ionization mode and 3.2 kV in positive ionization mode. Automatic gain control was set at 3E6 loads and 250 ms maximum injection time, 70,000 resolutions, and a scan time of 2.5 scans/s. The content of coumaric acid in HEBD was previously verified and was 5% in the dry extract, as Costa et al. [14] described.

### 2.3. In Vivo Trials

#### 2.3.1. Animals

All experiments were carried out following the International Standards and Ethical Guidelines on Animal Welfare and the ARRIVE guidelines and were previously approved by the Ethics Committee on the Use of Animals of the University of Vale do Itajaí (038/18p and 024/20p). Swiss male mice (60 days old, 30–40 g) from the central vivarium of UNIVALI were used. The animals were acclimated to controlled conditions for at least seven days before experimental handling, with free access to feed and water, at 23°C and under a 12-hour light/dark cycle. The study was undertaken by using 37 animals distributed in different groups.

#### 2.3.2. Colitis Induction

The experimental colitis was induced by adding 3% dextran sulfate sodium (DSS) to the animals' drinking water for seven consecutive days, followed by five days in which they received drinking water without DSS, according to the used by Meurer et al. [19]. The vehicle group (water plus 1% Tween-80, 10 mL/kg, *n* = 6), the HEBD groups (3, 30, or 300 mg/kg, *n* = 6), and the p-coumaric acid group (15 mg/kg, *n* = 6) received orally the treatments once a day for twelve days. The treatments started simultaneously with DSS intake.

The animals were weighed daily, and the presence of rectal blood and fecal consistency was individually analyzed, and each parameter was assigned a score according to Utrilla et al. [20], and these data were used to calculate the disease activity index (DAI). A noncolitis group (naïve group, *n* = 7) was monitored throughout the experiment and did not receive DSS in the drinking water but was treated with a vehicle. Finally, to verify whether the extract was able to change the behavior of the mice, an experiment was also carried out in noncolitis mice receiving HEBD (300 mg/kg, p.o).

#### 2.3.3. Histological and Histochemical Evaluation

On the 13^th^ day after the treatments, all animals were euthanized, and the colon was removed, measured, and weighted. A segment of approximately 0.5 cm was fixed in a solution composed of 85% alcohol, 10% formaldehyde, and 5% acetic acid. Subsequently, these samples were dehydrated, cleared, and embedded in paraffin. Sections of 5 *μ*m were obtained, deparaffinized, rehydrated, and submitted to staining in eosin-hematoxylin for morphological analysis. The histological damage was performed according to Utrilla et al. [20] and Camuesco et al. [21], considering epithelial loss, edema, cellular infiltration, goblet cells depletion, and the condition of the crypts.

The mucin levels were quantified through histochemical analysis on the sections obtained as described above using the periodic acid Schiff's (PAS) technique, which stained glycoproteins like mucins in pink. After the PAS procedure, the slides were observed under an optical microscope at 400 × magnification and photographed to quantify the mucin levels using the ImageJ® software, according to Pereira et al. [22].

#### 2.3.4. Biochemical Analyses

Samples from each mouse's colon, cortex, and hippocampus were homogenized with 200 mM potassium phosphate buffer (pH 6.5). Then, this homogenate was used to determine glutathione reduced (GSH) in accordance with Sedlak and Lindsay [23] and lipoperoxides (LOOH) as described by. The remaining material was centrifuged again at 9000 g for 20 minutes. The activity of glutathione S-transferase (GST), superoxide dismutase (SOD), and catalase (CAT), and the levels of reactive oxygen species (ROS), interleukin (IL)-4, and tumor necrosis factor (TNF) levels were determined in the supernatant, and the precipitate was used to determine myeloperoxidase (MPO) activity according to Bradley and collaborators [24] and modified by De Young and collaborators [25]. The SOD activity was determined based on the inhibitory capability of autoxidation of pyrogallol [26], whereas the determination of CAT and GST activities was according to the methods described by AEBI [27] and Habig et al. [28], respectively. A mouse cytokine kit acquired of BD Biosciences (Franklin Lakes, New Jersey, USA) was used to estimate TNF and IL-6 levels by enzyme-linked immunosorbent assay following the manufacturer's instructions. The Bradford method was adopted using bovine serum albumin (0.125 to 1.0 mg/mL) as standard to determine the protein concentration in each sample.

#### 2.3.5. Behavioral Tests

Behavioral tests were undertaken on the 11^th^ and 12^th^ days of the treatments to measure anxious and depressive behavior, respectively. Three different experimental models were used to assess depressive and anxious-like behavior in mice: elevated plus maze (EPM), tail suspension test (TST), and open field test (OFT).

The effects of HEBD in depressive-like behavior of health and colitis mice were evaluated using the TST following Steru et al. [29]. On 11^th^ day of treatments, the animals were suspended by the tail 30 cm above the floor using adhesive tape (1 cm from the tip of the end). The time of mouse immobility was recorded (in seconds) during the last 4 min of a 6-min session.

The spontaneous locomotor activity of mice was evaluated in the OFT to determine whether HEBD promotes changes in the locomotory activity of health or colitis mice. This test was performed on the 11^th^ day. Animals were individually placed in an acrylic box (50 × 50 × 50 cm), with the floor being divided into 24 equal squares. The number of squares crossed with the four paws (crossing) and the rearing (number of times the rat raised on its hind legs) was recorded in a 6-min session, similarly to.

On the 12^th^ day from the beginning of the treatments, the anxiety-like behavior of health and colitis mice was measured using the EPM (40 cm length, 10 cm width, and 50 cm height). Each mouse was placed in the central area of the maze facing one of the open arms, and all movements were recorded by the observer for 6 min [30]. The closed arms were enclosed by a black wall 20 cm in height.

### 2.4. Statistical Analysis

Analyses were performed using the GraphPad Prism 7.0 program (San Diego, USA). When applicable, statistical analysis was performed using one-way or two-way analysis of variance (ANOVA), followed by Bonferroni post-test. The results were presented as means ± standard error of means (S.E.M). Values of *p* < 0.05 were considered significant.

## 3. Results

### 3.1. Effect of HEBD and p-Coumaric Acid on Weight Loss and DAI Score in DSS-Induced Colitis Mice

**Figure 1:**
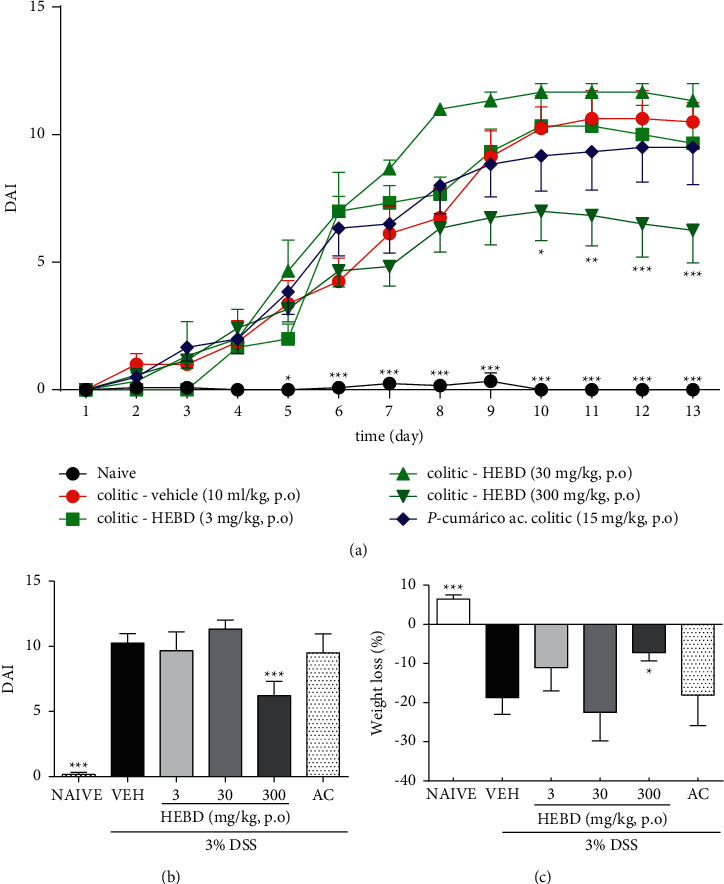
HEBD, but not p-coumaric acid, reduces colitis severity (panels A and B and prevents the weight loss (panel C in DSS-induced colitis mice). (a): DAI score over the entire treatment period. (b): DAI values at the 13^th^ after the beginning of treatments. (c): Weight loss at the 13^th^ after the beginning of treatments. Colitis mice were treated with the vehicle (VEH: water plus 1% Tween-80, 10 ml/kg, p.o) or hydroalcoholic extract of *B. dracunculifolia* (HEBD: 3–300 mg/kg, p.o). The naïve group was composed of noncolitis mice treated with the vehicle. Results are presented as the means ± S.E.M. (*n* = 6). One-way (panels B and C) or two-way (panel C) ANOVA followed by Bonferroni's multiple comparisons test.  ^*∗*^*p* < 0.05, ^*∗*^ ^*∗*^*p* < 0.05,  and  ^*∗*^ ^*∗*^ ^*∗*^*p* < 0.05 when compared to VEH.

The DAI score in the colitis mice treated with a vehicle reached media equal to 10.5 ± 1.05, whereas the colitis mice treated with HEBD (300 mg/kg, p.o) experienced a decrease in this value from 10^th^ day of the treatment, compared with the vehicle group (Figure 1(a)). Moreover, it was observed 39.2% of reduction in DAI score in the mice treated with HEBD (300 mg/kg, p.o) after the end of the treatment period, compared with the vehicle group (Figure 1(b)). However, the colitis group treated with p-coumaric acid showed no difference in the DAI score compared with the vehicle-treated colitis group (Figure 1(a)).

Regarding the weight loss of the animals, the difference between the naive group and the vehicle-treated colitis group was evidenced (*p* < 0.05), and the treatment with HEBD (300 mg/kg, p.o) prevented this weight loss compared with the vehicle-treated colitis group (Figure 1(c)).

### 3.2. Effects of HEBD and p-Coumaric Acid on Colon Length and in the Spleen, Liver, and Colon Weight of DSS-Induced Colitis Mice

The DSS intake reduced the colon length of the vehicle-treated colitis mice by 68% compared with the naive group (9.27 ± 0.70 cm) (Table 1). However, HEBD at the dose of 300 mg/kg did not prevent this decrease compared with the vehicle-treated colitis group. Interestingly, the HEBD at 3 mg/kg prevented by 16% the decrease in colon length compared with the vehicle-treated colitis group. The treatment with p-coumaric acid does not change the reduction of colon length in colitis animals (Table 1). Furthermore, the results also showed that there was a difference concerning the weight of the colon from the colitis mice treated with HEBD at doses of 30 and 300 mg/kg compared with the colitis mice treated with the vehicle or the naïve group (*p* < 0.05) (Table 1).

### 3.3. Effect of HEBD on Histopathological Changes in the Colon of DSS-Induced Colitis Mice

**Figure 2:**
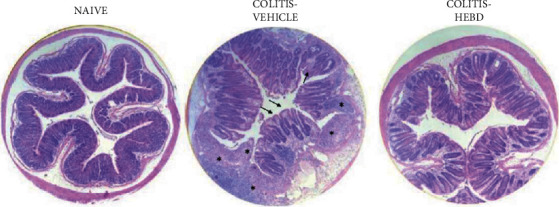
HEBD reduces on histological changes in colon tissue from DSS-induced colitis mice. NV: naive (noncolitis mice treated with the vehicle, water plus 1% Tween-80, 10 ml/kg, p.o); VEH: colitis mice treated with the vehicle; HEBD: hydroalcoholic extract of *B. dracunculifolia* (300 mg/kg). The asterisks indicate edema and inflammatory infiltrate, and black arrows indicate reduced villus height and crypt depth.

The colon histological architecture was intensely altered by the DSS intake in the vehicle group, presenting villus height and crypt depth reduced (points highlighted by black arrows) (Figure 2). In addition, intense edema in the lamina propria with significant inflammatory infiltrate was highlighted in the colitis mice treated with the vehicle (points indicated by asterisks). On the other hand, the treatment with HEBD (300 mg/kg) partially preserved the villi architecture and decreased the deleterious effect of DSS on intestinal tunics.

### 3.4. Effect of HEBD on DSS-Induced Changes in Colonic Mucin Levels

**Figure 3:**
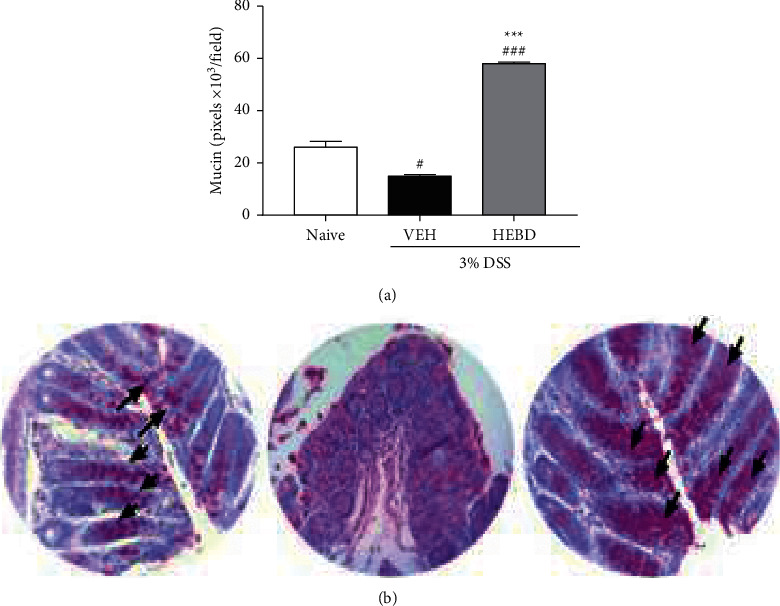
HEBD increased the mucin staining by PAS method in colon tissue from DSS-induced colitis mice. Colitis mice were treated with the vehicle (VEH: water plus 1% Tween-80, 10 ml/kg, p.o) or hydroalcoholic extract of *B. dracunculifolia* (HEBD: 300 mg/kg, p.o). (a) Results are presented as the means ± S.E.M. (*n* = 6), one-way ANOVA followed by Bonferroni post-test. ^###^*p* < 0.001 *and*^#^*p* < 0.05 compared with the naive (noncolitis) group (NV). ^*∗∗∗*^*p* < 0.0001 compared with the colitis group treated with the vehicle (VEH). (b) Microscopic representative image from each experimental. Arrows indicate positive points for mucin labeling (stained in pink).

**Figure 4:**
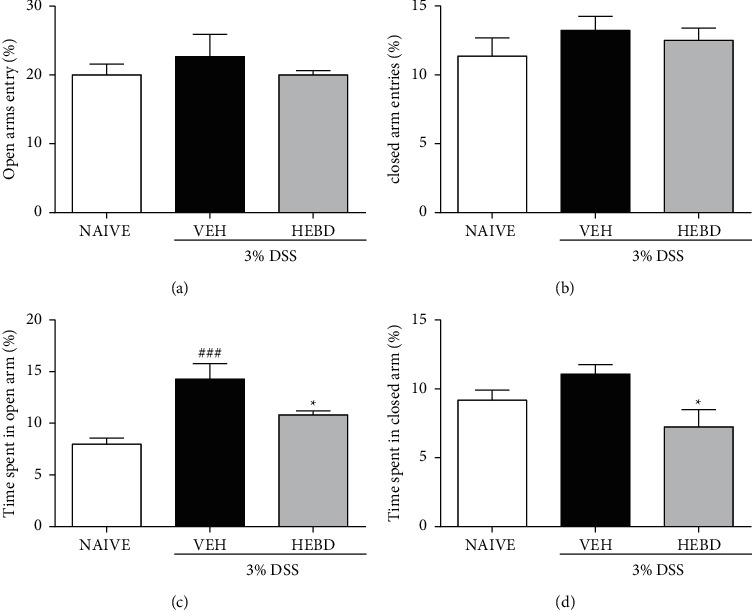
HEBD changes behavioral parameters of colitis mice in the EPM test. Colitis mice were treated with the vehicle (VEH: water plus 1% Tween-80, 10 ml/kg, p.o) or hydroalcoholic extract of *B. dracunculifolia* (HEBD: 300 mg/kg, p.o). Results are presented as the means ± S.E.M. (*n* = 6). One-way ANOVA followed by Bonferroni's post-test. ^###^*p* < 0.001 and ^#^*p* < 0.05 compared with the naive (noncolitis) group (NV). ^*∗*^*p* < 0.05 compared with the colitis group treated with the vehicle (VEH).

Representative images of each experimental group are shown in Figure 3(b). As quantified in Figure 4(a), the PAS staining in the colon of vehicle-treated colitis mice decreased by 46% compared with the noncolitis group (NV: 26 ± 4 × 103 pixels/field) (Figure 3(a)). In contrast, the treatment with HEBD (300 mg/kg) increased the mucin levels by 318% compared with the colitis mice treated with the vehicle.

### 3.5. Effect of HEBD on Behavioral of Colitis Mice Evaluated in the EPM Test

It was not observed a significant difference between the groups in the percentages of entry into the open or closed arms, respectively, Figures 4(a) and 4(b). However, the vehicle-treated colitis mice had an average of 14% in the length of stay in the open arms, which was significantly increased compared with the naive (noncolitis) group (*p* < 0.001). In contrast, the colitis group treated with HEBD presented a decrease in the length of stay in the open arms with the colitis group treated with the vehicle (Figure 4(c), *p* < 0.05), but with similar values to found in noncolitis mice (*p* > 0.05). The analysis of the length of stay in the closed arms was similar between the naive group and the colitis group treated with HEBD (*p* > 0.05); in contrast, the vehicle group had an increased average stay in the closed arms compared with the noncolitis mice (Figure 4(d), *p* < 0.05).

### 3.6. Effects of HEBD on Behavioral Parameters of Colitis Mice Evaluated in the TST

**Figure 5:**
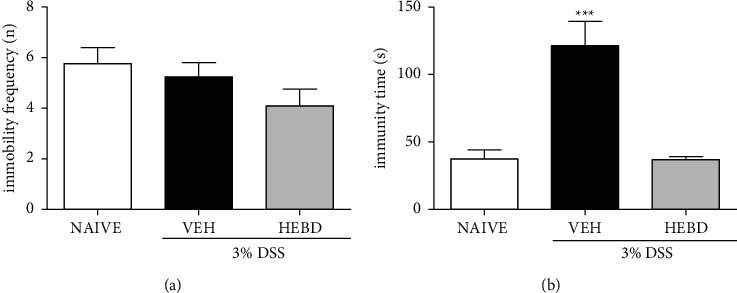
HEBD changes behavioral parameters of colitis mice in the TST. Colitis mice were treated with the vehicle (VEH: water plus 1% Tween-80, 10 ml/kg, p.o) or hydroalcoholic extract of *B. dracunculifolia* (HEBD: 300 mg/kg, p.o). Results are presented as the means ± S.E.M. (*n* = 6). One-way ANOVA followed by Bonferroni's post-test. ^*∗∗∗*^*p* < 0.001 compared with the noncolitis mice (NAIVE) or the colitis group treated with HEBD.

There was no statistical difference between groups in the frequency of immobility in the tail suspension test (Figure 5(a)), but it was possible to observe an increase in the immobility time in the vehicle-treated colitis mice compared with the naive group or the colitis group treated with HEBD (*p* < 0.001, Figure 5(b)).

### 3.7. Effects of HEBD on Behavioral Parameters of Colitis Mice Evaluated in the OFT

**Figure 6:**
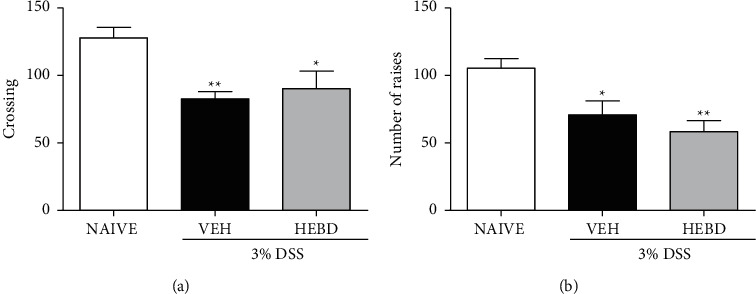
HEBD has not changed the behavioral parameters of colitis mice in the OFT. Colitis mice were treated with the vehicle (VEH: water plus 1% Tween-80, 10 ml/kg, p.o) or hydroalcoholic extract of *B. dracunculifolia* (HEBD: 300 mg/kg, p.o). Results are presented as the means ± S.E.M. (*n* = 6). One-way ANOVA followed by Bonferroni's posttest. ^*∗*^*p* < 0.05 and ^*∗∗*^*p* < 0.01 compared with the noncolitis mice (NAIVE).

The vehicle-treated colitis group showed a reduced number of crossings with the naive group (*p* < 0.01) (Figure 6(a)). Likewise, there was a significant difference between the naive and colitis groups treated with HEBD (*p* < 0.05) regarding the crossing activity. Further, the rearing activities of the colitis group treated with the vehicle (*p* < 0.05) or HEBD (*p* < 0.05) also were reduced compared with the naive (noncolitis) group (Figure 6(b)).

### 3.8. Effects of HEBD on DSS-Induced Biochemical Changes in the Colon of Mice

The GSH levels were decreased by 38% in the colitis group treated with the vehicle compared with the naïve (noncolitis) group, whereas the colitis group treated with HEBD (300 mg/kg, p.o) showed an increase of 51% in the GSH levels related to the vehicle-treated colitis group (Table 2).

The SOD activity was increased by 39% in the vehicle-treated colitis group compared with the naïve (noncolitis) group, and an increase of 107% in this parameter was evidenced in the colitis group that received HEBD (300 mg/kg) compared with the vehicle-treated colitis group (*p* < 0.01). Moreover, the CAT activity was decreased by 33% in the vehicle-treated colitis group compared with the naive (noncolitis) group, and the treatment with HEBD (300 mg/kg) increased by 41% the activity of this enzyme compared with the vehicle group (Table 2).

Regarding GST activity, no differences were observed between the vehicle-treated colitis group and the naïve (noncolitis) group, but there was a reduction of 32,4% in the GST activity in the colitis animals treated with HEBD (300 mg/kg). The LOOH and ROS levels did not differ between experimental groups (Table 2).

The MPO analysis showed a 53% increase in the vehicle-treated colitis group compared with the naive (*p* < 0.05). When comparing the colitis group treated with vehicle and the colitis group treated with HEBD, there is a 50% decrease (*p* < 0.01) in MPO levels in the colitis group treated with HEBD.

### 3.9. Effect of HEBD on Oxidative Stress Parameters in the Cortex and Hippocampus of Colitis Mice

The effect of HEBD on oxidative stress in the cortex and hippocampus is shown in Table 3. In the cortex, a decrease of 44% and 38% was observed in the colitis groups treated with the vehicle and HEBD in the GSH levels, with the naive group, respectively. There was a 24% decrease in the GSH content of the vehicle-treated colitis group in hippocampal samples compared with the naive group. On the other hand, when compared to the vehicle-treated colitis group, the treatment with HEBD increased by 44% of the GSH levels in the hippocampus of colitis mice.

In the cortex samples, a significant decrease in the SOD activity in colitis mice treated with the vehicle or HEBD was not observed compared with the naive (noncolitis) group. Similarly, the vehicle-treated colitis group had a 36% decrease in SOD activity in the hippocampus, compared with the naive group; however, the HEBD-treated colitis group showed an increase of 43% in the activity of this enzyme, compared with the vehicle-treated colitis mice.

It was not verified a significant difference in the CAT activity in both the cortex and the hippocampus of experimental groups (Table 3). Compared with the naive group, there was a 35% decrease in GST activity in the cortex and 22% in the hippocampus. In addition, the treatment with HEBD did not prevent the diminished GST activity in the cortex or hippocampus of colitis mice.

### 3.10. Effect of HEBD on TNF and IL-6 Levels in the Colon, Cortex, and Hippocampus of Colitis Mice

**Figure 7:**
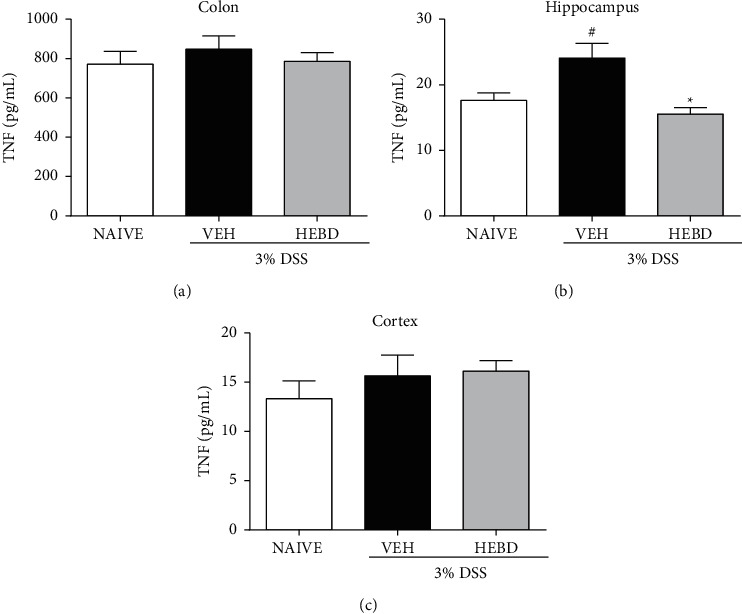
Effects of HEBD on TNF levels in colon (a), hippocampus (b), and cortex (c) of colitis mice. Colitis mice were treated with the vehicle (VEH: water plus 1% Tween-80, 10 ml/kg, p.o) or hydroalcoholic extract of *B. dracunculifolia* (HEBD: 300 mg/kg, p.o). Results are presented as the means ± S.E.M. (*n* = 6). One-way ANOVA followed by Bonferroni's posttest. ^#^*p* < 0.05 compared with naïve (noncolitis) group, and ^*∗*^*p* < 0.05 compared to colitis group treated with the vehicle.

There was an increase in TNF levels in the hippocampus of vehicle-treated colitis mice compared with the naive group (*p* < 0.05) (Figure 7(b)). A difference (*p* < 0.05) was also observed between the colitis group treated with HEBD and the colitis group treated with the vehicle, reducing TNF levels to values like the naive group. However, there was no difference between TNF levels in colon and cortex samples from different experimental groups.

**Figure 8:**
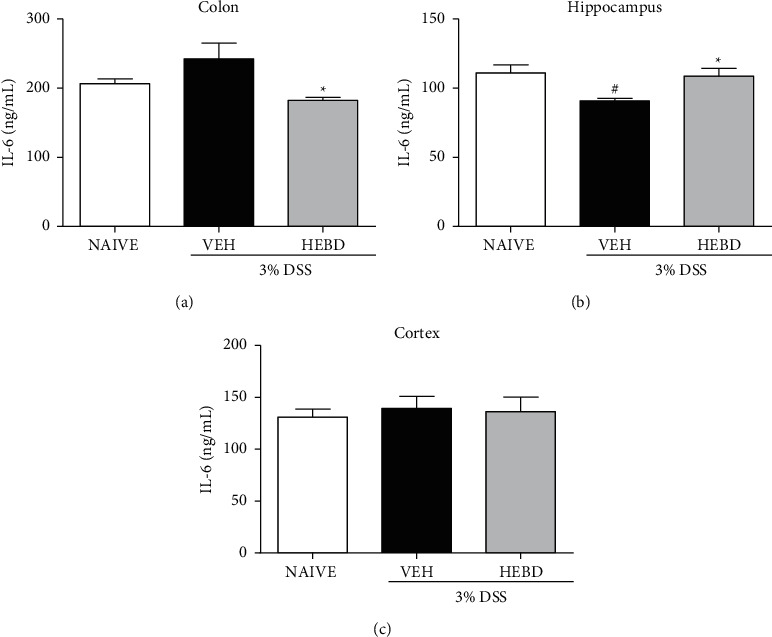
Effects of HEBD on IL-6 levels in colon (a), hippocampus (b), and cortex (c) of colitis mice. Colitis mice were treated with the vehicle (VEH: water, 10 ml/kg, p.o) or hydroalcoholic extract of *B. dracunculifolia* (HEBD: 300 mg/kg, p.o). Results are presented as the means ± S.E.M. (*n* = 6). One-way ANOVA followed by Bonferroni's posttest. ^#^*p* < 0.05 compared with naïve (noncolitis) group, and ^*∗*^*p* < 0.05 compared with colitis group treated with the vehicle.

Regarding IL-6 levels, the results observed in Figure 8(a) demonstrate a significant decrease (*p* < 0.05) in IL-6 levels in the colon of the colitis mice treated with the HEBD-treated compared with the vehicle-treated colitis group. Furthermore, there was a difference (*p* < 0.05) with increased levels of IL-6 in the hippocampus of colitis mice treated with HEBD and the naive group compared with the vehicle-treated colitis group (Figure 8(b)). Finally, there was no significant difference in IL-6 levels in the cortex of mice in any of the experimental groups (Figure 8(c)).

## 4. Discussion

The B. dracunculifolia has been used in the south and southeast of Brazil as a medicinal plant to treat inflammatory conditions; indeed, some authors have validated such use and described the pharmacological potential of preparations of this plant in models of inflammatory diseases [31, 32]. Nevertheless, a single experimental study reported the effects of the ethyl acetate extract from B. dracunculifolia against intestinal inflammation induced by TNBS in rats [13].

Strengthening the hypothesis that B. dracunculifolia is helpful as an ethnopharmacological resource in treating intestinal inflammation, this research described the hydroalcoholic extract of B. dracunculifolia (HEBD) attenuated the DSS-induced ulcerative colitis in mice. The DSS-induced ulcerative colitis model has the advantage of easy reproducibility and promotes colonic damage mediated by a predominantly TH2 immune response, like what occurs in humans affected by ulcerative colitis.

It is interesting to highlight that the results obtained by Cestari et al. [13] were achieved using an extract soluble in ethyl acetate and that the described effects of this study were obtained by the administration of a hydroalcoholic extract, obtained with water and alcohol, which is closest to the folkloric use of this plant. Moreover, corroborating with Cestari et al. [13], a reduction in the DAI values in colitis mice treated with HEBD at a dose of 300 mg/Kg was also observed, evidencing the pharmacological potential of HEBD to treat IBD.

The DAI is a parameter that assesses the remission or exacerbation of the disease during the experimental period and includes changes in stool consistency, body weight, and rectal bleeding [33]. In addition to the reduction in DAI, HEBD attenuates the weight loss of colitis mice, which also positively impacted DAI reduction in the HEBD-treated group. The main physical signs presented by individuals affected by UC are excessive weight loss leading to malnutrition [34], which compromises their quality of life [5]. Therefore, alternative treatments to mitigate weight loss are necessary.

The UC also compromises the colon's integrity, and the treatment with HEBD minimized the colon shortening in colitis mice, but only observed at the lowest dose tested, although this dose did not reduce the DAI. In addition, p-coumaric acid (15 mg/Kg), the major compound of B. dracunculifolia [35, 36], was not able to attenuate the DAI or prevent the decrease in colon length in colitis animals, which can be due to the dose administered chosen according to its content in the HEBD and the minimum effective dose of this extract in reducing DAI. p-Coumaric acid doses were established based on the concentration of this compound in HEBD. Luceri et al. [37] described that the p-coumaric acid at 50 mg/kg attenuated the intestinal inflammation induced by DSS due to its ability to suppress COX-2 expression and activity. Moreover, our research group showed the gastric healing effects of p-coumaric acid at a 15 mg/kg dose in rats [14]. Therefore, it is possible to infer that the p-coumaric acid may participate in the intestinal anti-inflammatory effects of the extract along with other constituents and not as an isolated mediator.

The main histological changes observed in the colon of mice after exposure to DSS were a decrease in mucin levels, changes in epithelial integrity with some necrosis areas, and neutrophil infiltration in the lamina propria and submucosa [38]. In fact, our slices were found edema and inflammatory infiltrate in the lamina propria, loss of crypts, and goblet cells, in addition to multifocal areas of erosions and ulcerations demonstrating significant tissue damage in the colon after exposure of the mice to DSS. Despite the ability of DSS to alter the colon histology integrity, it was possible to observe that the treatment with HEBD (300 mg/kg) partially preserved the villi architecture and reduced the deleterious effect of DSS on the intestinal tunics, corroborating with the diminished in the DAI values reached in these mice.

Epithelial mucins are mucus glycoproteins that make a layer that lines the gastrointestinal tract as a protective barrier that guarantees intestinal homeostasis acting as the first line of defense against xenobiotics, bacteria, viruses, fungi, or protozoa. The Goblet cells produce different types of mucins basally or in response to some stimulus (toxins, cytokines, neuropeptides, and growth factors). Our results showed that mucin levels in the colon of mice from the colitis group treated with the vehicle decreased. This reduction in mucin levels due to an inflammatory injury affects the mucus barrier. It increases intestinal permeability, which facilitates the passage of bacteria, microbial products, and toxins that cause damage to the epithelial cells, initiating a systemic inflammatory stimulus [39]. In contrast, the HEBD (300 mg/kg) prevented the decrease in mucin levels even in the intake of DSS. This effect occurred in the stomach of rats submitted to acetic acid–induced ulcer and treated with HEBD (300 mg/kg) [14], attesting that it favors the mucin secretion in the gastrointestinal tract mucosa and can play a protective factor during injurious processes in these tissues.

Dorofeyev et al. [39] described that in patients with UC and Crohn's disease, there was a depletion in mucin expression, compromising its protective function in the colon, and Liu et al. [40] showed the relationship with intestinal diseases and reduction in MUC2 expression. These data corroborate our results because colitis mice that received vehicle had greater DAI and more significant depletion in colonic mucin levels, while colitis animals treated with extract showed a reduction in DAI in parallel to mucin preservation.

In addition to the action of HEBD against intestinal damage due to DSS-induced colitis, we also investigated the neuroprotective effect of the extract in these colitis mice because of a relationship between IBD and neuropsychiatric comorbidities [41]. Byrne et al. [42] assessed the prevalence of anxiety and depression in patients with IBD; they found a high prevalence of these diseases in those populations and demonstrated that the active phase of the disease was significantly associated with an increased risk of depression and anxiety. A depressive-like behavior was observed in vehicle-treated colitis mice, evidenced by the increase in the immobility time in the TST. It was not observed in the group treated with HEBD.

Rivet-Noor [43] reviewed the evidence about the relationship between disruption of intestinal mucins and depression and addressed that stress can modify the delicate mucus-microbiome balance, initiating dysbiosis and ultimately leading to depression. Moreover, conducted a clinical with patients suffering from major depressive disorder (MDD) investigating pro-inflammatory pathways related to the “likable bowel” hypothesis associated with MDD, defined as an intestinal translocation of bacterial and an abnormal toll-like receptor-mediated immune response initiating a systemic inflammatory state, microglial activation, and neuroinflammation. Therefore, the similar behavior to the healthy group found in colitis mice treated with HEBD may be due to the preservation of the integrity of the intestinal barrier promoted by the extract or other effects that mitigate inflammatory events in the central nervous system.

The debility state of colitis mice treated with the vehicle might interfere with the data obtained. Therefore, the animals were evaluated in the OFT to verify the impact of locomotor activity on the obtained data. Furthermore, in the EPM test, the colitis group treated with the vehicle had a higher average stay in both open and closed arms when compared to the naive group or HEBD colitis group. Indeed, there was a reduction in exploratory activity in the colitis mice treated with the vehicle; however, the colitis group treated with HEBD also showed reduced in the crossing and rearing number, which could mean that although the extract reduced the severity of colitis, a reduction in mobility in this group remains but not to the degree that it impacts the data obtained in the TST and EPM test. Besides, the extract administration in healthy animals did not promote any behavior change.

The excessive production of reactive oxygen species (ROS) is related to the development of colitis, and endogenous antioxidant defenses, especially GSH, play an essential role in the attenuation of oxidative stress that is related to cytotoxicity, cell death, and inflammatory process due to intestinal inflammation [44]. In parallel to the increase in intestinal mucin, the HEBD also increased GSH levels and SOD activity in the colon. In agreement with these data, Mariano et al. [45] found that hydroalcoholic extract of Brazilian green propolis increased colonic GSH and SOD activity in the colitis mice, keeping these antioxidants at levels like those found in the noncolitis group.

Guimarães et al. [35] investigated the effects of the glycolic extract of B. dracunculifolia against oxidative stress in the liver of rodents. They attributed its beneficial effects to its antioxidant capacity, which is related to the compounds present in the extract, mainly phenols, and flavonoids. The SOD acts to convert the superoxide anion into hydrogen peroxide, and CAT, in turn, neutralizes it in water [46]. In addition to the increase in SOD activity, the colitis mice treated with HEBD showed an increase in CAT activity, demonstrating the favor of antioxidant defenses mediated the HEBD actions in the colon.

The central nervous system (CNS) is highly susceptible to oxidative stress, and the interactions that occur in the intestinal microbiota can also influence the oxidative state of the CNS [47]. In parallel to attenuating the colonic oxidative stress, the HEBD treatment also increased the levels of GSH and the SOD activity in the hippocampus from colitis mice. In contrast, the extract did not prevent GSH depletion and SOD activity reduction in the cortex of colitis animals, evidencing specificity in the site of action.

An increase in MPO activity, a biomarker of neutrophil infiltration, was found in the colitis group treated with the vehicle; however, the HEBD treatment reduced this inflammatory marker. Previously, Costa et al. [14] demonstrated that the hydroalcoholic extract of Brazilian green propolis also reduced the MPO activity in gastric tissues and attributed the intestinal anti-inflammatory effects of this propolis to its ability to inhibit TH1 differentiation in a model of colitis induced by TNBS.

Our results also agree with Cestari et al. [13] using TNBS-induced UC in rats treated with an extract of B. dracunculifolia. Another study also associated the anti-inflammatory effect of the HEBD with its ability to inhibit cyclooxygenase (COX)-2, which is often increased in inflammatory processes [48]. Moreover, the inflammatory process associated with colitis increases the production of inflammatory cytokines, mainly TNF and IL-6 [18]. TNF plays an essential role in the progression of UC as it stimulates immune system cells to produce different chemokines, cytokines, and prostaglandin E2, in addition to ROS and RNS, which culminates in an increase in intestinal permeability and consequent recruitment of neutrophils to the inflamed colonic tissue [49]. However, unexpectedly, in our study, we did not observe a significant difference in TNF levels in the colon of colitis mice.

As TNF, IL-6 is also involved in the inflammatory process related to colitis and is associated with the regulation of the adaptive immune response [49]. In the study by Wang et al. [50], in which the authors also used a murine model of ulcerative colitis induced by DSS and azoxymethane, they found significantly higher levels of TNF-*α* and IL-6 in colitis mice compared with the control group. These data partially corroborate our results, although no difference was observed in TNF-*α* levels, IL-6 levels were significantly lower in the colon of colitis mice treated with HEBD, confirming once more the intestinal anti-inflammatory capacity of this extract.

A recent study discussed that high serum levels of IL-6 and TNF may be related to depressive symptoms [51]. A significant increase in TNF levels in the hippocampus, but not cortex, of vehicle-treated colitis mice was observed, along with a depressive-like behavior assessed by the TST in this experimental group. Interestingly, Do and Woo [52] described that the hippocampus is more susceptible to neuroinflammation in animal models of DSS-induced colitis, and later, this inflammation can progress to other brain regions. Furthermore, it has been shown that rats with colitis have increased excitability in the hippocampal lamina.

Opposite to TNF, there was a reduction in IL-6 levels in the hippocampus of vehicle-treated colitis mice. However, although IL-6 is a pro-inflammatory cytokine, it can also act in regenerative or anti-inflammatory activities, depending on the type of signaling [53]. However, it is essential to note that animals treated with extract had similar levels of IL-6 to healthy animals in both the colon and hippocampus.

## 5. Conclusions

In conclusion, HEBD by oral route reduced the severity of colitis due to its ability to minimize weight loss and colon shortening and preserve the colonic histology. The adequate level of colon mucin in colitis mice treated with HEBD, along with the reduction in inflammation and oxidative stress in the colon of treated animals, placed this extract as a natural resource to treat intestinal inflammatory diseases, corroborating the medicinal use of B. dracunculifolia as anti-inflammatory.

Furthermore, HEBD has also been shown to be effective in preventing behavioral changes in colitis mice, mainly attenuating depressive behaviors that may have been mediated by the inhibitory effects in inflammatory and oxidative markers in the hippocampus in parallel to the degree of preservation in the intestinal barrier in colitis mice that received the extract.

## Figures and Tables

**Table 1:** Effects of HEBD and *p*-coumaric acid on colon length and in the spleen, liver, and colon weight of DSS-induced colitis mice.

Groups	Dose	Colon length (cm)	Colon weight (g/100 g)	Spleen weight (g/100 g)	Liver weight (g/100 g)
Naive	10 mL/Kg	9.3 ± 0.7	0.06 ± 0.01	0.05 ± 0.01	0.50 ± 0.10
Colitis—vehicle	10 mL/Kg	6.3 ± 0.9^d^	0.07 ± 0.01	0.08 ± 0.01^c^	0.41 ± 0.01
Colitis—HEBD	3 mg/Kg	7.4 ± 0.8^a^	0.08 ± 0.01	0.06 ± 0.01	0.43 ± 0.01
Colitis—HEBD	30 mg/Kg	6.0 ± 0.8^d^	0.11 ± 0.01^b,c^	0.08 ± 0.01	0.53 ± 0.10
Colitis—HEBD	300 mg/kg	6.9 ± 0.8^d^	0.09 ± 0.01^b,c^	0.08 ± 0.0^c^	0.44 ± 0.01
*p*-Coumaric acid	15 mg/kg	6.9 ± 1.5^d^	0.07 ± 0.01	0.07 ± 0.01	0.43 ± 0.01

**Table 2:** Effect of HEBD on biochemical parameters in colon of colitis mice.

	Naive	Colitis vehicle	Colitis HEBD
GSH	831.7 ± 67.5	509.5 ± 65.7^b^	772.3 ± 84.7^b^
ROS	2315 ± 347	2079 ± 624	1813 ± 155.3
LOOH	3.8 ± 0.3	3.6 ± 0.8	1.9 ± 0.1
SOD	18.7 ± 3.6	11.4 ± 1.5^c^	23.6 ± 6.4^b^
CAT	6.1 ± 1.4	4.0 ± 1.2^c^	5.7 ± 1.2^a^
GST	3.3 ± 0.1	3.2 ± 0.3	2.1 ± 0.6^b,d^
MPO	0.03 ± 0.01^a^	0.04 ± 0.01	0.03 ± 0.01^b^

Colitis mice were treated with the vehicle (VEH: water plus 1% Tween-80, 10 ml/kg, p.o) or *B. dracunculifolia* hydroalcoholic extract (HEBD: 300 mg/kg, p.o). GSH: reduced glutathione (*μ*g/g tissue). ROS: reactive oxygen species (fluorescence units). LOOH: lipid hydroperoxide (mmol/g of tissue). SOD: superoxide dismutase (U/mg protein). CAT: catalase (mmol/mg protein). GST: glutathione S-transferase (mmol/mg protein). MPO: myeloperoxidase: m O.D/mg protein. Results are presented as the means ± S.E.M. (*n* *=* 6). One-way ANOVA followed by Bonferroni's posttest. ^a^*p* < 0.05 and ^b^*p* < 0.01 vs. vehicle-treated colitis and ^c^*p* < 0.05 and ^d^*p* < 0.01 vs. naive (noncolitis) groups.

**Table 3:** Effect of HEBD on oxidative stress parameters in the cortex and hippocampus of colitis mice.

	Naive	Colitis vehicle	Colitis HEBD	Naïve	Colitis vehicle	Colitis HEBD
	Cortex			*Hippocampus*		
GSH	419.7 ± 49.0	234.2 ± 143^a^	261.3 ± 8.5^a^	352.9 ± 36.6	268.6 ± 31.7^a^	386.9 ± 53.2^b^
SOD	4.1 ± 0.4	2.2 ± 0.1^a^	2.4 ± 0.5^a^	4.3 ± 0.4	2.7 ± 0.1^a^	3.9 ± 0.3^b^
CAT	0.7 ± 0.1	0.7 ± 0.2	0.5 ± 0.1	0.7 ± 0.2	0.8 ± 0.2	1.0 ± 0.2
GST	9.0 ± 0.1	5.8 ± 0.5^a^	6.5 ± 0.4^a^	6.3 ± 1.1	4.9 ± 0.5^a^	4.6 ± 0.6^a^

Colitis mice were treated with the vehicle (VEH: water plus 1% Tween-80, 10 ml/kg, p.o) or *B. dracunculifolia* hydroalcoholic extract (HEBD: 300 mg/kg, p.o). GSH: reduced glutathione (*μ*g/g tissue); SOD: superoxide dismutase (U/mg protein); CAT: catalase (mmol/mg protein); and GST: glutathione S-transferase (mmol/mg protein). Results are presented as the means ± S.E.M. (*n* = 6). One-way ANOVA followed by Bonferroni's posttest. ^a^*p* < 0.05 vs. vehicle-treated colitis and ^b^*p* < 0.05 vs. naïve (noncolitis) group.

## Data Availability

The data that support the findings of this study are available from the corresponding author, LMS, upon reasonable request.
